# CD207^+^ Langerhans cells constitute a minor population of skin-derived antigen-presenting cells in the draining lymph node following exposure to *Schistosoma mansoni*

**DOI:** 10.1016/j.ijpara.2006.10.007

**Published:** 2007-02

**Authors:** Supeecha Kumkate, Gavin R. Jenkins, Ross A. Paveley, Karen G. Hogg, Adrian P. Mountford

**Affiliations:** Department of Biology, The University of York, York, UK

**Keywords:** Langerhans cell, CD207, *Schistosoma*, Helminth, Vaccine

## Abstract

Infectious cercariae of *Schistosoma mansoni* gain entry to the mammalian host through the skin where they induce a transient inflammatory influx of mononuclear cells. Some of these cells have antigen-presenting cell function (MHCII^+^) and have been reported to migrate to the skin-draining lymph nodes (sdLN) where they have the potential to prime CD4^+^ cells of the acquired immune response. Here, in mice exposed to vaccinating radiation-attenuated schistosome larvae, which induce high levels of protective immunity to challenge infection, we describe the parasite-induced migration of Langerhans cells (LCs) from the epidermal site of immunisation to the sdLN using a specific monoclonal antibody that recognises langerin (CD207). CD207^+^ cells with dendritic morphology were abundant in the epidermis at all times and their migration into the dermis was detected soon after vaccination. All CD207^+^ LCs were MHCII^+^ but not all MHCII^+^ cells in the skin were CD207^+^. LCs migrated from the dermis in enhanced numbers after vaccination, as detected in dermal exudate populations recovered after in vitro culture of skin biopsies. Elevated numbers of CD207^+^ LCs were also detected in the sdLN from 24 h to 4 days after vaccination. However, compared with other dermal-derived antigen-presenting cells that were CD207^−^MHCII^+^ or CD207^−^CD11c^+^, the relative numbers of CD207^+^ cells in the dermal exudate population and in the sdLN were very small. Furthermore, the migration of CD207^+^ cells after exposure to ‘protective’ radiation-attenuated, compared with ‘non-protective’ normal cercariae, was similar in terms of numbers and kinetics. Together, these studies suggest that CD207^+^ LCs are only a minor component of the antigen-presenting cell population that migrates from the epidermis and they are unlikely to be important in the priming of protective CD4^+^ cells in the sdLN.

## 1 Introduction

Schistosomiasis is a parasitic disease affecting over 200 million people in many parts of the developing world but as yet an effective vaccine has not been developed. Infection of the mammalian host occurs by penetration of the skin by free-swimming larvae present in contaminated water. Once infective cercariae locate host skin, they enter the stratified epidermis, aided by the secretion of enzymes from the parasites’ acetabular glands (Salter et al., 2002). The schistosome larvae then move through the skin until they reach the epidermal–dermal basement membrane, which acts as a barrier prior to their entry into the dermis. Although the timing of parasite migration through the skin is not agreed (McKerrow and Salter, 2002; Curwen and Wilson, 2003), studies in the mouse suggest that penetration of the epidermis occurs within 30 min and the majority of larvae remain in the skin for at least 40 h (Wheater and Wilson, 1979). The parasites then exit the skin via blood venules or lymphatic vessels, en route to the lungs and then the blood vessels of the hepatic portal system (*Schistosoma mansoni*) or the bladder vesical plexus (*S. haematobium*).

Irrespective of the final site of parasitisation, a skin phase of migration is common to all species of schistosomes and can be analysed effectively using a murine model (Stirewalt, 1974). Although penetration of the skin usually only elicits transient dermal inflammation (Incani and McLaren, 1984; Hogg et al., 2003a), repeated exposure can result in cercarial dermatitis, which in the case of avian schistosomes is characterised by a Th2-associated immediate hypersensitivity reaction (Kourilova et al., 2004). Nevertheless, the skin also provides a logical site to target in the development of an anti-larval vaccine (Mountford and Trottein, 2004). In this context, radiation-attenuated (RA) *S. mansoni* cercariae consistently elicit high levels of protection characterised by the induction of Th1-associated immune responses (Hewitson et al., 2005). Analysis of the skin exposure site after vaccination reveals focal inflammatory reactions composed of a heterogeneous array of infiltrating leukocytes orchestrated by a cascade of chemokines and cytokines (Riengrojpitak et al., 1998; Hogg et al., 2003a,b). Significantly, secretion of IL-12p40 by in vitro-cultured skin biopsies from vaccinated mice was more sustained compared with its transient production by skin biopsies from mice exposed to normal (non-irradiated) larvae (Hogg et al., 2003a). Of those cells that had the capacity to migrate from the skin to the skin-draining lymph nodes (sdLNs) many were MHCII^+^. Moreover, the majority of the dermal exudate cells (DECs) that were positive for intracellular IL-12p40 also expressed surface CD11c and/or F4/80, indicating that they were dendritic cells (DCs) and/or macrophages (Hogg et al., 2003a).

The fact that a large proportion of larvae remain in the epidermis for greater than 24 h, and that cells with the potential for antigen-processing presentation (i.e. were MHCII^+^) migrated from the skin, raised the question as to whether epidermal Langerhans cells (LCs) were a significant feature of the dermal immune response leading to the induction of protective immunity by RA schistosomes. Epidermal LCs are efficient antigen-presenting cells (APCs) in that they capture foreign antigens and process them into antigenic fragments (Romani et al., 2003). They also acquire the ability to migrate and so can transport antigen to the sdLN where initiation and differentiation of the adaptive T-cell response occurs (Schuler and Steinman, 1985; Steinman and Swanson, 1995). Although LCs share a number of surface markers/receptors with other APCs including DCs (e.g. CD11c), a transmembrane lectin named langerin (CD207) unique to LCs was recently discovered (Valladeau et al., 2000). CD207 is abundantly expressed by epidermal LCs and freshly isolated LCs but is down-regulated upon stimulation via CD40 and in vitro culture (Valladeau et al., 2002; Stoitzner et al., 2003). It is also an important marker to enable the tracking of LC migration and distinguishing them in the sdLN where several different populations of DCs are present (Salomon et al., 1998; Henri et al., 2001; Stoitzner et al., 2003). In this manner, we can determine the importance of LCs relative to other dermal DCs in initiation of the immune response to schistosome antigens in the sdLN.

In the context of parasite infection, LCs may be an important APC in the development of cutaneous immunity to *Leishmania major* (Moll et al., 1993; Moll, 2003), since LCs carry a substantial load of *L. major* promastigotes (Baldwin et al., 2004), and *Leishmania*-exposed LCs are thought to be the major cell type responsible for the production of functional IL-12 (von Stebut et al., 1998). Moreover, LCs directly transport ingested *L. major* from the site of infection to the T-cell areas of sdLN (Arnoldi and Moll, 1998), although others have more recently questioned the relative importance of LCs in the priming of acquired immunity against *L. major* (Filippi et al., 2003; Lemos et al., 2004; Ritter et al., 2004). The parasitic helminth *Brugia malayi* also induces the migration of LCs from human epidermis (Semnani et al., 2004). In contrast, little is known about how LCs respond to cutaneous schistosome infection. Recently, schistosomes were shown to delay the migration of LCs (defined as MHCII^+^) from the skin, mediated by PGD_2_ produced by parasites themselves (Angeli et al., 2001). However, it is not known whether LCs play a significant role in the priming of the Th1-acquired response to the well studied RA schistosome vaccine in the mouse, although LCs have been implicated in the induction of protection in a guinea pig model (Sato and Kamiya, 1995).

In the present study, we used an antibody against CD207 (Stoitzner et al., 2003), specifically to track LC migration from the epidermis to the sdLN following percutaneous exposure of mice to RA schistosome larvae. We report for the first time that schistosomes induce the migration of CD207^+^ LCs from the epidermis to the sdLN but in this site they represent only a minor population of skin-derived APCs after schistosome exposure.

## 2 Materials and methods

### 2.1 Host and parasite

Anaesthetised female C57BL/6 strain mice (8–10 weeks old) were exposed to between 200 and 500 optimally irradiated (20-krad; ^60^Co source) or normal (non-irradiated) cercariae of *S. mansoni* via each pinna as described previously (Mountford et al., 2001).

### 2.2 Preparation of tissues

The skin site of exposure and the sdLN (auricular) were obtained from groups of mice at 0, 8, 16 and 24 h, and on days 2, 4 or 8 after parasite exposure. Tissues were prepared for histology, culture in vitro, or analysis by flow cytometry as described below. For conventional histology, 5 μm traverse sections of wax-embedded pinnae (fixed in 10% buffered formalin) were stained with H&E. For immunohistological analysis, pinnae and the sdLN were embedded in OCT compound (H&E Ltd., Nottingham, UK) and snap frozen in 2-methylbutane on liquid N_2_. Cryosections were then cut (7–14 μm; Leica Microsystems cryostat, Germany) and placed on poly-l-lysine-coated glass slides (Superfrost Plus; Invitrogen, Paisley, UK) before fixing in 3.7% paraformaldehyde. Epidermal sheets were obtained by splitting pinnae and floating them on 0.5 M ammonium thiocyanate in PBS at 37 °C for 30 min (Gunn et al., 1999). The sheets were then peeled from the underlying dermis and rinsed in PBS prior to fixing in 3.7% paraformaldehyde on ice for 15 min.

For analysis of cells either on cytospins or by flow cytometry, single suspensions were obtained as follows. Epidermal cells were isolated by floating split pinnae on 0.8% trypsin-PBS (Roche Applied Science, Germany) in 24-well culture plates (Nalgene, Nunc) at 37 °C for 30 min (Koch et al., 1992; Stoitzner et al., 2003). Trypsin digestion was stopped with RPMI medium containing low endotoxin 10% FCS (Seralab, Oxon.UK), 2 mM l-glutamine, 200 U/ml penicillin and 100 mg/ml streptomycin (RPMI/10). The epidermal sheet was then removed, disrupted on a vortex mixer for 10 s to break up the cell aggregates and then filtered through steel gauze to yield a single cell suspension.

Cells that emigrate from the skin (DEC) were isolated as described previously (Mountford et al., 2001; Hogg et al., 2003a,b). Briefly, pinnae were split and floated on 0.5 ml RPMI/10 in 24-well hydrophobic culture plates (Greiner Labortechnik, Frickenhausen, Germany) for 18 h at 37 °C in 5% CO_2_. Cells which spontaneously detach from the split pinnae were harvested, enumerated and resuspended in PBS containing 0.5 mM CaCl_2_, 0.5 mM MgCl_2_ and 0.1% BSA. The sdLN were digested with collagenase D (0.5 mg/ml; Roche, Germany)/DNAse (0.02 mg/ml; Sigma) at room temperature for 20 min (Wang et al., 1999) and the stromal fragments disrupted before treating with 0.5 mM EDTA for 5 min to disrupt DC-T cell complexes.

### 2.3 Antibodies

Rat anti-mouse Langerin mAb (clone F929F3, rat IgG; provided by Dr. Saeland, Schering-Plough, Dardilly, France) was used to identify CD207^+^ LCs (Valladeau et al., 2002; Stoitzner et al., 2003) followed by goat anti-rat Ig conjugated with Alexafluor 488 (MolecularProbes, Leiden, The Netherlands) or Allophycocyanin (Caltag-Medsystems, Towester, UK). This primary mAb specifically reacts with cytoplasmic epitopes of CD207, therefore immunolabeling was performed using an intracellular staining protocol including 0.1% saponin at each incubation step. To stain cell surface markers, biotinylated antibodies were as follows: anti-CD4 (clone H129.19 Pharmingen, BD Biosciences), anti-MHC II (I-a^b,d^, clone B21.2, Caltag-MedSystems) or anti-CD11c (clone HLA-3, Pharmingen) followed by streptavidin-conjugated Texas-Red (Vector Laboratories, Peterborough, UK), APC or fluorescein isothiocyanate (FITC) (Caltag MedSystems). Appropriate isotype-matched non-specific antibodies were used throughout to establish control level background staining. Parasites in the skin were localised using a polyclonal rabbit antiserum against solubilised schistosome larvae (Mountford et al., 1995) and probed using goat anti-rabbit antibody conjugated to Alexa 594 (Molecular probes).

### 2.4 Immunofluorescent staining

Fixed sections of pinnae and sdLN or epidermal sheets were first treated with Avidin–Biotin blocking kit (Vector Labs) to block non-specific binding due to endogenous biotin. Sections were subsequently incubated with relevant primary unlabelled, or biotin-labelled antibodies, as described above, alongside irrelevant isotype-matched control antibodies. Tissue sections were then probed as appropriate with fluorescent-conjugated streptavidin, or secondary mAbs prior to mounting with VectorShield (Vector Labs). Alternatively, aliquots of relevant cell suspension were spun at 500 rpm for 5 min (Cytospin 2, Shandon, UK) and labelled as above for CD207 and MHCII.

### 2.5 Skin sensitisation with FITC

Naïve mice were painted with 25 μl 1% FITC (Sigma) preparation dissolved in 1:1 acetone:dibutylphalate (Sigma) on the dorsal side of both ears, or with control vehicle only (acetone:dibutylphalate). Tissues were removed at 24 h for analysis.

### 2.6 Analysis of cells in tissue sections

Tissue sections were viewed using a Nikon Labophot fluorescent microscope (Nikon Corp, Tokyo, Japan) and photographed using a Nikon Coolpix 995 (Nikon Corp) with subsequent analysis performed with Adobe Photoshop version 6. Confocal images were acquired on a Zeiss Axiovert 200 M with an LSM 510 META system. All multicolour samples were imaged sequentially and controls showed no bleed-through. Images were collected using the LSM 510 software v3.2 and copied using Zeiss Image Browser. The number of CD207^+^ cells was determined per image, the frequency was converted to LC/mm^2^ and the results expressed as mean + SEM of a minimum of 10 images.

### 2.7 Flow cytometric analysis of LCs in epidermal, dermal exudate and sdLN cell suspensions

Aliquots of up to 5 × 10^5^ cells from the epidermis, DEC and sdLN were briefly fixed in 1% formaldehyde in PBS and then permeabilised with 0.1% saponin in PBS containing 2% FCS. Cells were blocked with 2 μl rabbit IgG (Vector Labs) prior to staining for intracellular CD207 as above. To label surface markers, CD207-stained cells were washed twice with saponin-free PBS plus 2% FCS before an excess of rat IgG (Sigma) was added to block residual-free antibody binding sites (Stoitzner et al., 2003). Cells were then labelled with phycoerythrin conjugated anti-MHCII^+^ (M5/114.15.2, PharMingen) or anti-CD11c mAb. Phenotypic data were acquired using a Cyan flow cytometer (Dakocytomation, UK) and analysed with Summit^®^ software.

### 2.8 Statistics

Data were compared using the Student’s *t* test. (∗∗∗, *P* < 0.001; ∗∗, *P* < 0.01; ∗, *P* < 0.05; non-significant, *P* > 0.05). Arithmetic means ± SEM are shown. Data shown are representative of two to four experimental repeats.

## 3 Results

### 3.1 LCs are present in the epidermis but at reduced numbers after parasite exposure

**Fig. 1:**
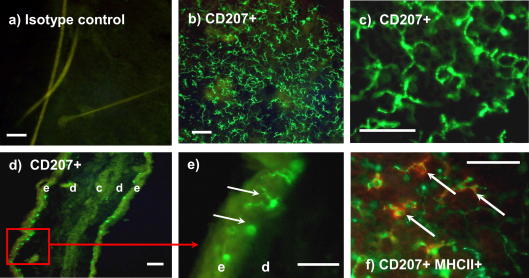
Localisation of CD207^+^ cells on epidermal sheets (a–c and f) and transverse sections of naïve mouse pinna (d and e). Cells were labelled with rat IgG control (a), rat anti-CD207 probed with anti rat Alexafluor 488 (green; b–f) and biotinylated anti-MHCII probed with streptavidin Texas-Red (f). Scale bars indicate 10 μm. Key: e, epidermis; d, dermis; c, cartilage.

Analysis of epidermal sheets of naïve mice probed with anti-CD207 mAb specifically revealed the presence of LCs with a characteristic dendritic morphology (Fig. 1b and c). The cells were evenly distributed throughout the epidermal sheet with the dendrites reaching out to access most of the tissue area. CD207^+^ LCs were distributed along the suprabasal layer of the epidermis but not within the dermis of naïve mice as observed in transverse sections through the mouse pinna (Fig. 1d and e). All of the CD207^+^ cells were also positive for surface MHC II (Fig. 1f). However, while CD207 was observed even in the remote thin part of the dendrites, MHC II molecules were sparse in the cell extremities and tended to be concentrated around the main cell body.

**Fig. 2:**
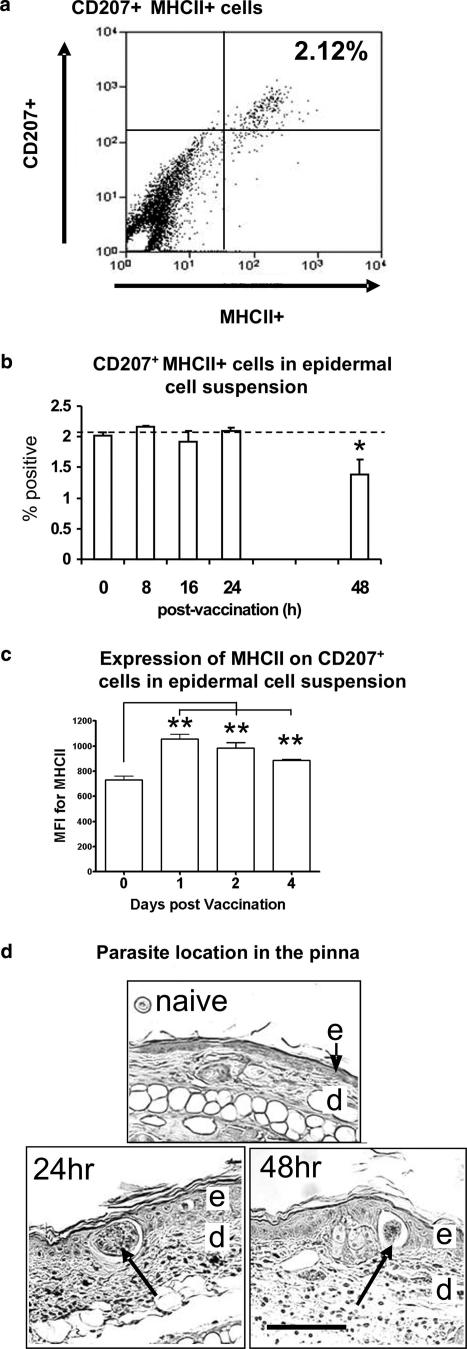
CD207^+^ cells in epidermis at times after vaccination. (a) Representative flow cytogram of CD207^+^MHCII^+^ cells in epidermal cell suspensions at time 0. (b) The proportion of CD207^+^MHCII^+^ cells at times 0, 8, 16, 24 and 48 h post vaccination, as defined by flow cytometric analysis. Horizontal dotted line indicates the value in naïve (day 0) mice ^∗^*P* < 0.05. (c) Relative expression level of MHCII on CD207^+^MHCII^+^ cells shown as the mean fluorescence intensity (MFI) at days 0, 1, 2 and 4. Bars are means of three to six pinnae ± SEM ^∗∗^*P* <0.01. (d) Location of parasites (arrowed) in the pinnae at 24 and 48 h showing increased cellularity of both epidermis and dermis. Scale bar indicates 50 μm. Key: e, epidermis; d, dermis.

Upon visual enumeration of CD207^+^ cells in epidermal sheets of mice exposed to RA larvae, there were 40% fewer cells on day 4 compared with naïve controls (day 4 = 270 ± 33 *cf*. day 0 = 454 ± 77; *P* < 0.05, data not shown). However, due to concerns about the subjectivity of LC enumeration in epidermal sheets, cell suspensions of the epidermis were also analysed by flow cytometry. In naïve tissues, approximately 2% of the total epidermal cell population was identified as CD207^+^MHCII^+^ (Fig. 2a and b); there were no CD207^+^MHCII^−^ cells detected. However, although the proportion of CD207^+^MHCII^+^ cells did not change at 8, 16 or 24 h, a significant 32% decrease (*P* < 0.05) was detected at 48 h when the LCs comprised only 1.4% of the total epidermal cell suspension (Fig. 2b). The expression of MHCII on CD207^+^ epidermal cells increased significantly within 1 day of vaccination (Fig. 2c) and remained significantly elevated out to day 4, although the level was declining to that detected in naïve mice. The decrease in the number of CD207^+^ cells in the epidermis followed an increase in the thickness and cellularity of both the epidermis and dermis at times when parasites were still present in the epidermis (Fig. 2d).

### 3.2 CD207^+^ LCs emigrate from the epidermis after parasite exposure

**Fig. 3:**
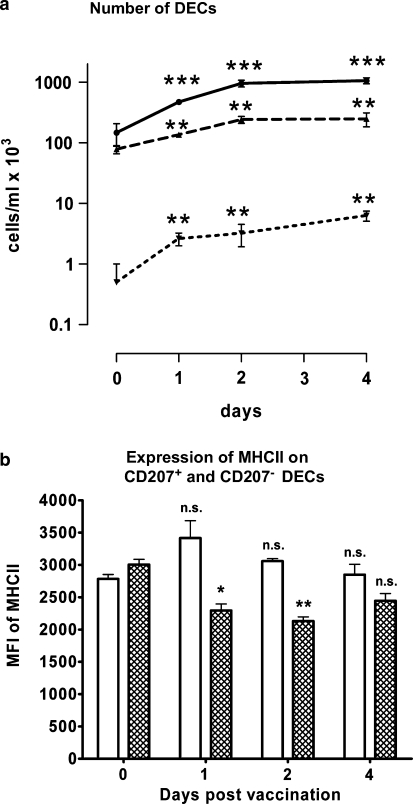
CD207^+^ cells in the dermal exudate population after vaccination. (a) Dermal exudate cell (DEC) numbers are shown for total (solid line), CD207^−^MHCII^+^ (dashed line) and CD207^+^MHCII^+^ (dotted line) cells at days 0, 1, 2 and 4. Lines are means of DEC populations from individual mice (*n* = 4), ±SEM. Significance is for the number of cells at times after vaccination compared with time 0. (b) Relative expression level of MHCII on CD207^+^MHCII^+^ DEC (open bar) and CD207^−^MHCII^+^ DEC (cross-hatched bar) shown as the mean fluorescence intensity (MFI) of MHCII staining on days 0, 1, 2 and 4. Bars represent mean of DEC from vaccinated mice (*n* = 3) ± SEM. Significance values are for CD207^+^ or CD207^−^ cells at different days compared with day 0 ^∗^*P* < 0.05, ^∗∗^*P* < 0.01.

The DEC population that spontaneously migrated from pinna biopsies during in vitro culture comprised a substantial number of MHCII^+^ cells but only a minority were also positive for intracellular CD207 (Fig. 3a). Between 15.1 ± 0.86% (day 2), 25.1 ± 1.51% (day 4) and 32.4 ± 2.83% (day 8) of the DEC population were MHCII^+^ but less than 1% (0.29–0.57%) of the total were CD207^+^. However, all CD207^+^ cells were MHCII^+^ since no CD207^+^MHCII^−^ cells were detected at any time point. Multiplication of these percentages by the total number of cells recovered at different times after vaccination show that the number of CD207^−^MHCII^+^ and CD207^+^MHCII^+^ DECs increased significantly by day 1 (both *P* < 0.01; Fig. 3a). This was followed by a consistent increase in both groups of cells at later time points. The numbers of CD207^−^MHCII^+^ cells in the DEC population was about 15-fold greater than the number of CD207^+^MHCII^+^ cells at all times (*P* < 0.01–0.001). There is a hint that the extent of MHCII expression on migrating CD207^+^MHCII^+^ cells increases after vaccination (i.e. day 1 *cf*. day 0) but this was not significant (Fig. 3b). In comparison, the level of expression of MHCII on CD207^−^MHCII^+^ cells decreases significantly on days 1 and 2 post vaccination (Fig. 3b). Therefore, CD207^+^ cells appear to have the potential to be better APCs than CD207^−^ cells, although they appear much less frequently (Fig. 3a).

**Fig. 4:**
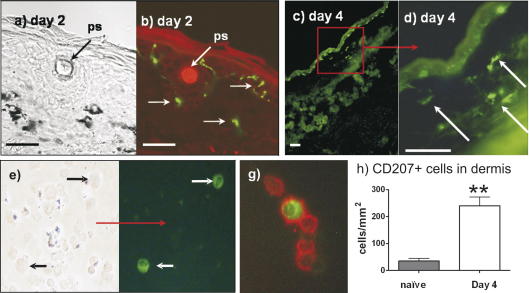
Emigration of CD207^+^ cells into the dermis. Transverse section of pinna on day 2 (a) shown under bright field demonstrating a parasite in the epidermis. Confocal image of the same section (b) showing CD207^+^ cells (green) in relation to the parasite (red). Focal grouping (c and d) of emigrating CD207^+^ cells in the dermis on day 4. Cytospins of dermal exudates cell (DEC) population under (e) phase contrast and (f) stained for CD207. The majority of DECs (g) are MHCII^+^ (red) and only a minority are CD207^+^ (green). Scale bars = 25μm. Mean number ± SEM of CD207^+^ cells per mm^2^ dermis (h) of naïve mice or on day 4. Significance values are for day 4 *cf*. day 0 ^∗^*P* < 0.05.

Histological examination of CD207^+^ cells in the dermal tissue confirmed that while they were found exclusively in the epidermis of naïve mice (Fig. 1d and e), a small number were observed in the dermis on days 2 (Fig. 4b) and 4 (Fig. 4c and d) post vaccination. A schistosome larva observed in the epidermis on day 2 is associated with CD207^+^ cells in the underlying region of the dermis (Fig. 4a and b). CD207^+^ cells were also evident in the epidermis but were absent in the immediate vicinity of the parasite. CD207^+^ cells in the dermis tended to be observed in groups and were not evenly scattered, suggesting a focal response to parasite exposure (Fig. 4c and d). There was evidence that the number of CD207^+^ cells in the epidermis immediately adjacent to the groups of dermal-located cells was reduced but this was not possible to enumerate. The scarce presence of CD207^+^ cells in the DEC population (Fig. 3a) was confirmed visually by staining of DECs on cytospins revealing occasional CD207^+^ cells (Fig. 4e and f), which were always MHCII^+^ but most MHCII^+^ DEC were not CD207^+^ (Fig. 4g). Visual enumeration of CD207^+^ cells in the dermis shows there to be over 5-fold more cells in the dermis on day 4 after vaccination than in naïve mice (*P* < 0.01; Fig. 4h).

### 3.3 CD207^+^ cells selectively accumulate in lymph nodes draining inflamed skin

**Fig. 5:**
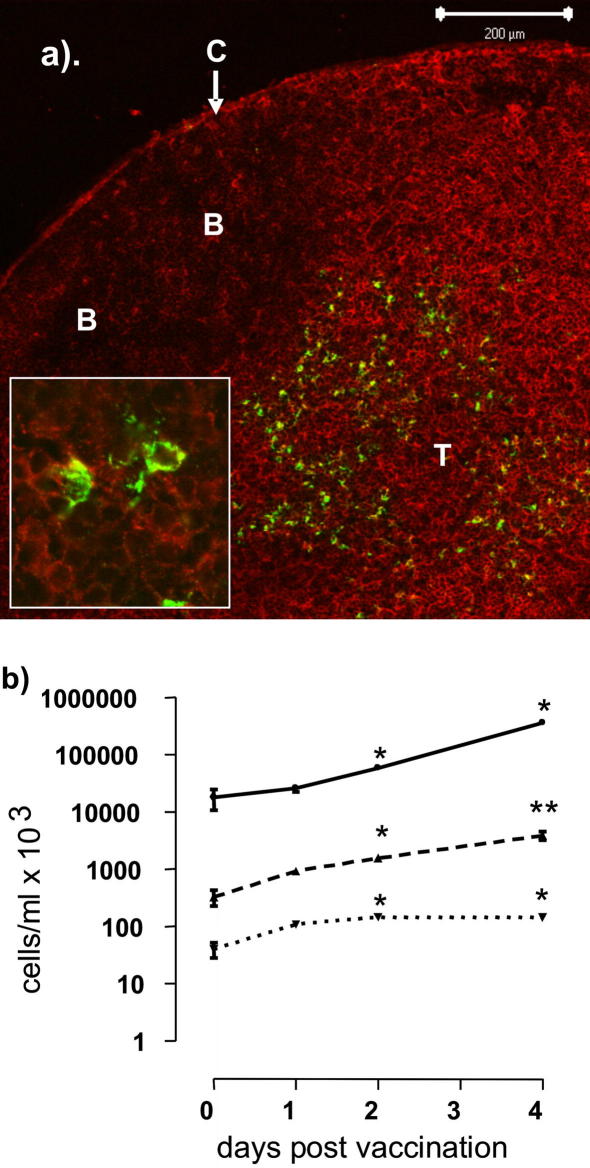
CD207^+^ cells are induced to migrate to the skin-draining lymph node (sdLN) after vaccination. (a) CD207^+^ cells (green) are located in CD4^+^ cell (red) rich inner paracortical areas of the sdLN (200× magnification). (Inset) Close up view of CD207^+^ cells in close contact with CD4^+^ cells (600×). Key: C, LN capsule; B, B cell zone/germinal centre in marginal cortex; T, T cell zone in the inner paracortical region. (b) Numbers of total (solid line), CD207^−^MHCII^+^ (dashed line) and CD207^+^MHCII^+^ (dotted line) cells in the sdLN at days 0, 1, 2 and 4 post vaccination. Lines are means of sdLN populations from individual mice (*n* = 4), ±SEM. Significance values are for cell numbers at days after vaccination compared with day 0 ^∗^*P* < 0.05, ^∗∗^*P* < 0.01.

Of the three peripheral lymph nodes (LNs) located in the neck/throat region, only the auricular LN contained CD207^+^ cells (Fig. 5a and b). Virtually no CD207^+^ cells were observed in the adjacent cervical and axillar LN, or the distant mesenteric LN draining the large intestine (data not shown). Significantly, CD207^+^ cells were restricted to the T-cell zone in the inner paracortical areas and none were found in the B cell follicles of the marginal cortex (Fig. 5a). At higher magnification, CD207^+^ cells can be observed with extended processes in close apposition to CD4^+^ cells in the T-cell zone (Fig. 5a inset). The overall increase in the number of cells in the sdLN after vaccination was mirrored by a significant increase in the number of CD207^+^MHCII^+^ cells by days 2 and 4 (*P* <  0.05; Fig. 5b). An increase in the number of CD207^+^MHCII^+^ cells was also apparent by day 1, although this was not statistically significant. The profile of CD207^+^MHCII^+^ cell numbers was similar to the increase in the overall number of CD207^−^MHCII^+^ cells which were 8-fold (day 1) to 26-fold (day 4) more abundant.

### 3.4 Dermal APCs are preferentially recruited to the sdLN after sensitisation

**Fig. 6:**
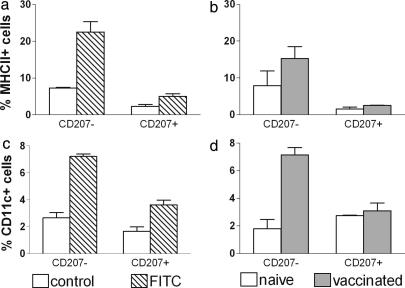
Relative migration of CD207^+^, CD11c^+^ and MHCII^+^ cells to the skin-draining lymph node (sdLN) after skin priming. Migration of Langerhans cells (CD207^+^MHCII^+^ or CD207^+^CD11c^+^), dermal dendritic cells (CD207^−^CD11c^+^) and other antigen-presenting cells (CD207^−^MHCII^+^) into the sdLN (a and b) 24 h after sensitising the epidermis with fluorescein isothiocyanate (FITC) (hatched bar) versus empty vehicle control (open bar), or (c and d) 48 h after exposure to vaccinating parasites (shaded bars) versus naïve (open bar). Bars are means of sdLN (*n* = 4), ±SEM.

Since the number of CD207^+^ cells recruited to the sdLN after parasite exposure seemed very limited, we made a comparison with cell migration following a conventional skin sensitising agent known to cause the migration of LCs (Douillard et al., 2005; Kissenpfennig et al., 2005). To increase the sensitivity of detection, the percent-positive events were gated to the largest and most granular cells (LGC; represents approximately 10% of the total sdLN cell pool). By day 1 after painting the epidermis with FITC, the proportion of MHCII^+^ LGC that were CD207^+^ doubled from 2.3% in mice painted with control vehicle only, to just over 5% (Fig. 6a). The proportion that were CD207^−^ increased 3-fold from 7.3% to over 20%. By comparison, after parasite exposure the proportion of MHCII^+^ LGCs that were CD207^+^ increased only 1.5-fold from 1.6% to 2.5%, and the proportion of CD207^−^ cells increased by just 1.9-fold to 15.3% (Fig. 6c).

A similar picture emerges from analysis of cells probed with the conventional DC marker CD11c (Fig. 6b). The proportion of CD207^+^ cells increased by 2.2-fold to 3.6% in the sdLN of mice painted with FITC, while the proportion of CD207^−^ cells which will be dermal DCs (not LCs) increased 2.7-fold to over 7%. In comparison, the proportion of CD207^+^CD11c^+^ cells in the sdLN of mice exposed to schistosome larvae only increased by 1.1-fold to 3.1% but the proportions of CD207^−^CD11c^+^ cells again increased by 4-fold to reach over 7% (Fig. 6d).

### 3.5 Migration of LCs in response to RA and normal schistosomula

**Fig. 7:**
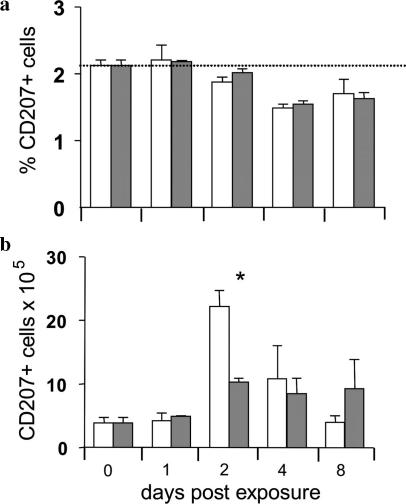
Migration of CD207^+^MHCII^+^ cells out of the epidermis into the skin-draining lymph node (sdLN) of vaccinated and infected mice. (a) Proportion of CD207^+^MHCII^+^ cells in the epidermal cell suspension and (b) total numbers in the sdLN on days 0, 1, 2, 4 and 8 p.i. (open) or after vaccination (shaded). Bars are means of sdLN (*n* = 4), ±SEM. Horizontal line is the value in naïve mice. Significance is for the difference in vaccinated compared with infected mice ^∗^*P* < 0.05.

Since RA schistosome larvae exhibit retarded migration from the skin compared with normal larvae (Mountford et al., 1988), it is possible that the emigration of CD207^+^ LCs from the skin to the sdLN in response to these two forms of parasite differs. A reduction in the CD207^+^ cell population in the epidermis was evident by 48 h following exposure to RA larvae (Fig. 2a). However, in a direct comparative analysis, the numbers of CD207^+^ cells observed in the epidermis following exposure to normal and RA larvae were shown to be similar and not significantly different (*P* > 0.05; Fig. 7a). In this experiment, significantly reduced numbers of epidermal CD207^+^ cells were apparent only by day 4 (*P* < 0.01); the reduction was still evident by day 8 after parasite exposure in both groups of mice.

Analysis of the CD207^+^ population in the sdLN revealed that both RA and normal larvae initiated similar changes to the proportions of CD207^+^MHCII^+^ cells at all time points. This equated to between 2% and 3% of the large granular cell population as noted earlier (Fig. 6a). However, when the cellularity of the sdLN in the two groups of mice was taken into account, it became evident that the number of LCs in the sdLN of mice exposed to normal larvae was significantly greater than in the sdLN of mice exposed to RA larvae on day 2 (*P* < 0.05; Fig. 7b). The number of CD207^+^MHCII^+^ in mice immunised with RA larvae was only greater than in infected mice by day 8, although this difference was not significant.

## 4 Discussion

For the first time to our knowledge, we show that schistosome larvae induce the migration of CD207^+^ LC from the site of immunisation to the sdLN. However, although LCs have the potential to be important antigen-processing and -presenting cells, the limited numbers of migrants in the context of a substantial antigenic insult (i.e. the immunising larvae) makes it unlikely that they have a dominant role in priming the early CD4^+^ immune response in the draining lymphoid tissue.

Using the rat mAb F929F3 which specifically recognises a cytoplasmic epitope of CD207 (langerin; Valladeau et al., 2002), we confirm that LCs are a relatively common cell within the epidermis and they form an evenly distributed network across the epidermal surface (Valladeau et al., 2002; Stoitzner et al., 2003). Although LCs express other surface antigens (e.g. MHCII, CD205 and CD11c), these are also expressed by other cell types such as DCs and macrophages, and only CD207 is restricted to LCs. LCs have been reported to occur in a ratio of 1:15 relative to keratinocytes (Mulholland et al., 2006); in the present study using flow cytometric enumeration, the CD207^+^ cell population of the naïve epidermis appeared to be less frequent, being approximately 2% of the total epidermal cell population. In our immunohistochemical studies, CD207^+^ cells locate in the suprabasal layer of the epidermis, above the epidermal basement membrane which also acts as a barrier to onward parasite migration (Wheater and Wilson, 1979). Indeed, multi-photon imaging of LCs positions them approximately 3 μm above the basement membrane (Mulholland et al., 2006), ideally located to come into contact with schistosome larvae. We confirmed that all CD207^+^ cells in the epidermis are also MHCII^+^. Interestingly, the relative level of MHCII expression by CD207^+^ cells in the epidermis is enhanced after vaccination, probably in response to acute phase cytokines such as IL-1β which we know is released soon after vaccination (Hogg et al., 2003a). This reinforces the notion that they could be efficient antigen-processing cells with the ability to present antigen. The dendrites of the LC population can be seen to extend across and access virtually the entire epidermal layer acting as a well-placed sentinel to detect and pick up antigens from incoming pathogens. The dendrites are thought to exhibit dSEARCH (dendrite surveillance extension and retraction cycling habitude) activity and the cells can also move laterally in response to inflammatory stimuli (Nishibu et al., 2006). Therefore, the intricate network of LCs in the epidermis should be able to detect most, if not all, of the percutaneously applied schistosome larvae used to immunise the murine host in our studies. Indeed, the much greater size of schistosome larvae makes it highly likely that they would come into contact with at least one LC.

Exposure of the skin to *S. mansoni* larvae induced the migration of some CD207^+^ cells from the epidermis within 24 h, which was confirmed by histological examination of the immunisation site, demonstrating that limited numbers of CD207^+^ cells migrate from the epidermis into the underlying dermis. The migratory response of CD207^+^ cells appeared to be focal with cells detected as groups in the dermis rather than as an even scatter. In sections where a parasite was detected at the epidermal basement membrane (e.g. at day 2), the CD207^+^ cells were located ‘downstream’ of the parasite and it is probable that these cells were migrants on their way to dermal lymphatic vessels leading to the sdLN. There was no evidence that CD207^+^ cells migrate laterally towards schistosomes since, rather than an accumulation, there was a relative absence in the immediate vicinity of epidermally located parasites. In tissue sections where a parasite was not detected, groups of CD207^+^ cells were also detected in the dermis. This might be due to the parasite having already left the skin site or the parasite had migrated out of the plane of view. Microscopy studies of living tissues using multi-photon technology should establish the precise inter-relationship of LCs and parasites in the epidermis.

Analysis of the number of CD207^+^ cells in the epidermis initially supports the notion of parasite-induced migration of LCs from the epidermis since fewer were counted in epidermal sheets 4 days after parasite exposure. However, flow cytometric enumeration of digested epidermal cell suspensions revealed that the number of CD207^+^ cells only declined in the epidermis by days 2–4 and the numbers detected within the first 48 h were similar to those in naïve mice. In this respect, it is possible that CD207is down-regulated by inflammatory stimuli such as lipopolysaccharide and ligation of CD40 (Takahara et al., 2002), such that LCs are no longer identified as CD207^+^ and might be underestimated, but this could be just a feature of in vitro culture (Stoitzner et al., 2003). On the other hand, CD207 is believed to be expressed during the migration of LCs (Kamath et al., 2002; Stoitzner et al., 2003) and characterises mature rather than immature LCs (Valladeau et al., 2000). The phenomenon of LC migration blockade following exposure to *S. mansoni* was originally reported by Angeli et al. (2001). They showed that the migration of epidermal LCs identified only by the expression of MHCII^+^ was inhibited following schistosome infection; this was transient and mediated by the production of parasite-derived prostaglandin D2 which specifically disrupted TNFα-induced migratory potential (Angeli et al., 2001). Furthermore, the active parasite component was later described to be a molecule with glutathione-*S*-transferase activity by acting on the D prostanoid receptor 1 (Herve et al., 2003). In part, our studies support the reported inhibition of LC migration following parasite exposure. However, through use of the anti-CD207 mAb as a specific marker for LCs, as distinct from other dermal-derived MHCII^+^ cells, we are also able to show that some LCs are able to migrate out of the epidermis within the first 48 h and begin to accumulate in the sdLN as early as 24 h after parasite exposure. Combined, our results suggest that the total number of LCs increases after parasite exposure, possibly from a population of Gr-1^hi+^ monocytes (Ginhoux et al., 2006), and that while some are retained in the epidermis, a proportion successfully leave within the first 24–48 h and migrate via the dermis to accumulate in the sdLN. Another conclusion that might be made from our results, is that migrating CD207^+^MHCII^+^ DECs express greater amounts of MHCII after vaccination than CD207^−^MHCII^+^ DECs. This suggests that migrating CD207^+^ LCs have a greater capacity to efficiently present antigen upon arrival at the draining LN than migrating CD207^−^ cells, such as dermal DCs or macrophages.

The detection of CD207^+^ cells in the sdLN of naïve mice was at first surprising but the staining was clearly specific since CD207^+^ cells were not observed in other LNs such as the mesenteric LN. This supports the view that the CD207^+^ cells in the sdLN observed in our studies were not the minor subset of lymphoid origin recently identified as CD11c^hi^, CD8α^+^, CD11b^lo^ which can be located in the mesenteric LN and spleen; rather they are of the CD11c^lo^, CD8α^lo^, CD11b^hi^ subset of epidermal origin (Douillard et al., 2005). The presence of CD207^+^ cells in substantial numbers in the sdLNs of naïve mice most likely reflects their traffic under normal conditions in the absence of an inflammatory stimulus (Stoitzner et al., 2005). Nevertheless, elevated numbers of CD207^+^MHCII^+^ cells were clearly detected in the sdLN after parasite exposure which progressively increased by day 4. Although this increase was marked, it was overshadowed by a far greater increase in the numbers of MHCII^+^ cells that were CD207^−^. Indeed, the number of MHCII^+^CD207^−^ cells were 10 times more frequent than CD207^+^ cells; this does not just reflect a proportionally greater increase of MHCII^+^ B lymphocytes in the sdLN but also an increase in CD207^−^CD11c^+^ cells which increased 16-fold compared with a 3.1-fold increase in CD207^+^CD11c^+^ cells. The increase in cells with APC function is likely to be cells originating from the skin in response to signalling via CCR7 (Ohl et al., 2004) but it is clear that only a small proportion are classical CD207^+^ LCs. The majority of APCs are likely to be macrophages and CD11c^+^ DCs, since both are abundant components of the dermis (Dupasquier et al., 2004). Our data comparing the migration of CD207^+^ and CD207^−^ cells in response to a contact sensitising agent (FITC) or RA schistosome larvae, support the view that, under inflammatory conditions, MHCII^+^ macrophages and/or dermal DCs are the most abundant cells to migrate from the skin to the sdLN and that CD207^+^ LCs are only a minor constituent despite expressing greater levels of MHCII. This difference in cell numbers may be a function of their relative migratory dynamics since data accrued from intravital imaging of LC migration in vivo using transgenic mice with the enhanced green fluorescent protein under the *CD207* gene, proposes that CD207^+^ LCs are slower to migrate to the sdLN than dermal DCs (Kissenpfennig et al., 2005; Kissenpfennig and Malissen, 2006).

Although we have evidence that CD207^+^ and other cells with APC function migrate from the skin to the sdLN, we have no proof that they process parasite antigen in the skin prior to departure or that they present the same antigen to CD4^+^ cells in the sdLN. They may even cross-present antigen to other APCs (Stoitzner et al., 2006) in the skin prior to migration of the latter to the sdLN. Furthermore, both parasites and parasite antigen drain directly to the sdLN (Mountford et al., 1988) independent of any migrating APCs, and antigen could be presented by resident APCs in situ. Unfortunately, it has not yet proven possible to stably transfect invasive cercariae with a plasmid expressing fluorescent probe(s) to allow visual detection of antigen uptake by MHCII^+^ cells. Another unresolved issue is whether CD207^+^ LCs in the DEC population are IL-12p40^+^ as originally proposed (Hogg et al., 2003a). We have been unable to convincingly co-detect intracellular IL-12p40 and intracellular CD207 using the currently available mAbs (Gavin Jenkins, unpublished data). IL-12 production by LC may actually be down-regulated, as reported to occur in susceptible strains of mice after infection with *L. major* (Moll et al., 2002), although intriguingly, ligation of CD154 on LCs is reported to stimulate IL-12p40 production (Sugaya et al., 2005) and may explain the up-regulation of IL-12p40 release by skin biopsies following anti-CD40 mAb treatment of CD154^−/−^ mice exposed to the RA schistosome vaccine (Hewitson et al., 2006).

The rationale for analysing the migration of CD207^+^ LCs in our model of parasite immunisation was that LCs would be important in the priming of a CD4^+^ mediated immune response in the sdLN, which ultimately would lead to the development of a protective immune response (Sato and Kamiya, 1995; Hogg et al., 2003a; Hewitson et al., 2005). Therefore, we compared the dynamics of CD207^+^ cell migration in mice exposed to protective RA larvae or non-protective normal parasites. The vast majority of normal parasites exit from the skin by day 8, whilst nearly one third of RA larvae remain (Mountford et al., 1988). However, no difference was determined in the rate of CD207^+^ cell migration out of the exposure site, despite the prolonged antigenic stimulus in the pinnae of vaccinated mice. Similarly, no difference was observed in the proportion of CD207^+^ cells between the sdLN from the two groups of mice. Therefore, the presence of CD207^+^ cells in the sdLN does not correlate with the previously reported enhanced CD4^+^ cell proliferation and Th1 cytokine production that is a feature of mice exposed to the RA schistosome vaccine (Hogg et al., 2003b; Hewitson et al., 2005).

On the other hand, it is possible that CD207^+^ cells, despite expressing slightly greater amounts of MHCII, do not have an immune priming role but regulate events in the sdLN, or cross-present antigen to other APCs (Stoitzner et al., 2006). In certain viral infection models, CD8α^+^ dermal DCs and not LCs have the major antigen-presenting function (Allan et al., 2003; Zhao et al., 2003). Moreover, several studies of *L. major* infection undermine the role of LCs as being responsible for priming the CD4^+^-mediated acquired immune response and instead highlight the role of dermal DCs (Filippi et al., 2003; Lemos et al., 2004; Ritter et al., 2004). In this context, we recently reported that molecules released by the invading schistosome larvae in the first few hours (i.e. equivalent to when the parasite is still in the epidermis) activate bone-marrow-derived DCs to become potent stimulators of Th2-type responses in vitro and in vivo (Jenkins and Mountford, 2005). These molecules may have an immunomodulatory function via their constituent glycans through the ligation of toll-like receptor 4 leading to the production of IL-10 (Jenkins et al., 2005a,b). Indeed, it is reported that *B. malayi* larvae act on LCs to depress MHCII expression and gene transcripts for cytokines associated with antigen presentation leading to a decreased ability to prime CD4+ cells responses (Semnani et al., 2004). It is therefore possible that CD207^+^ LCs have an, as yet, unappreciated role in modulating the immune response to schistosomes distinct from its presumed role as an APC. Indeed, recent observations on the dynamics and functions of CD207^+^ cells fuel speculation in a number of different inflammatory situations that LCs have a regulatory, rather than stimulatory, role (Itano et al., 2003; Douillard et al., 2005; Villadangos and Heath, 2005; Kissenpfennig and Malissen, 2006).
